# 

**Published:** 1894-06-02

**Authors:** 


**Notices of Births, Deaths, and Marriages** are inserted in this column at a charge of 2s. 6d. for an announcement not exceeding four lines.

**Notice to Advertisers and Subscribers.**

All Advertisements, Orders for Copies of The Hospital, and Business Communications should be addressed to **The Publisher, NOT TO THE EDITOR,** The Hospital, 428, Strand, W.C.

The Cost of Subscription, post free, is as follows:—Per week, 2½d.; per quarter, 2s. 8d.; per half-year, 5s. 3d.; per annum, 10s. 6d. Foreign:—Per Annum, 12s. 8d.; payable, in all cases, in advance.

### NOTICE TO CORRESPONDENTS.

All MS., Letters, Books for Review, and other matters intended for the Editor should be addressed The Editor, The Lodge, Porchester Square, London, W.

The Editor cannot undertake to return rejected MS., even when accompanied by stamped directed envelope.

**"Burdett's Hospital Annual," 1894.**

Fifth year of publication. 900 pp. Boards, gilt lettering. A most complete guide to British, Colonial, Indian, and American Hospitals, Dispensaries, Nursing and Convalescent Institutions, and Asylums. Price 5s. Published by The Scientific Press (Limited), 428, Strand, W.C.

**NOTICE.—The Index for Vol. XIV. (ending Sept. 30th, 1893) is on sale at the Office of this Journal, price 2½d., post free.**

### SCRAPS AND GLEANINGS.

The Hospital Sunday Fund at Newcastle amounted for total receipts to £4,475.

Extensive laundry premises are to be added to the South Eastern Hospital.

The Royal National Hospital for Consumption at Ventnor has received an anonymous donation of £250.

The Duke of York has consented to preside at the banquet to be held on June 1st for St. Mary's Hospital.

T.R.H. the Prince and Princess of Wales have consented to open the Poplar Hospital for Accidents on June 11th.

The 143rd annual report of the Royal Infirmary, Newcastle, shows that the income has exceeded the expenditure by £398.

The Duke of Fife presided at the annual meeting of the Children's Holiday Fund, Whitehall Rooms, Metropole Hotel, on May 15th.

By the death of Miss Sarah Harrison, which occurred at Wakefield on Monday, some £30,000 will become payable to the Clayton Hospital and other charities.

At the eleventh International Medical Congress at Rome Professor Virchow was presented with the distinguished order of Saint Maurice and Saint Lazarus.

The expenditure of the Birmingham Skin and Lock Hospital has been £1,997 for the past year, which is £152 more than last year, and £158 in excess of the income.

The Children's Hospital League, which has only been in existence for a period of 10 months, has raised £73 in favour of the funds of the New Hospital and Dispensary.

The annual ball held in favour of the funds of the Royal South Hants Infirmary has just taken place, and proved a great success, as well as a financial support to the Institution.

The Duke of Fife has kindly consented to preside at a meeting to be held at the Mansion House on July 5th, at four p.m., on behalf of the eight Homes for Working Boys in London.

Sir Algernon Borthwick, Bart., M.P., has kindly consented to preside at a festival dinner in aid of the Chelsea Hospital for Women, on Tuesday, June 26th, at the Hotel Metropole.

The Committee of the New Borough Hospital for Burton have accepted a tender for £7,900 for the erection of the buildings. The hospital will probably be completed in eighteen months' time.

A New paddle steamboat for the river ambulance service of the Metropolitan Asylums Board for the purpose of conveying patients from the metropolitan district to the hospital ships at Long Reach, has been launched.

The balance-sheet at the annual meeting of the Winter's Home and Hospital, Starbeck, showed a donation and subscription list to the amount of £300, patients having added another £200 to the funds of the institution.

The 13th annual meeting of the Norwood Cottage Hospital took place recently. Annual subscriptions to the same are reported at £407. The Samaritan Fund has enabled fourteen patients to be sent to Bexhill Convalescent Home with expenses paid.

The treasurer of the Aberdeen Royal Infirmary Corporation reports the receipt of a legacy of £300 to the funds of the institution by the late Mr. W. Simpson. The trustees of Mr. John Gordon's charitable fund have presented £75 to the same infirmary.

To the forward movement of the Birmingham Hospital Saturday Committee an interesting addition has been made in the establishment near Llandudno Junction of a Convalescent Home for Women, the opening ceremony of which took place on Saturday, May 5th.

At the annual meeting of subscribers to the Braintree and Bocking Cottage Hospital a fairly satisfactory financial statement was made, there being a balance to the good on the year's working accounts. A legacy of £250 was referred to from the late Miss Wakeham.

The Radcliffe Infirmary at Oxford has received a contribution of £500 from Mr. James Mason, of Eynsham Hall, which sum is to be applied in aid of the new building fund. Another £700 is required to complete the work of the new block, which, when finished, will cost £6,000.

During the last recorded month the National Society for the Prevention of Cruelty to Children investigated 1,446 complaints of cruelty. Of these cases 950 were warned, 162 were prosecuted, and 206 dealt with in other ways. The penalties inflicted were 29¼ years of imprisonment and £80 in fines.

The revenue for the North Ormesby Cottage Hospital shows an increase during the past year of £64, but owing to the increased number of in-patients the expenditure was much larger. During the period under review 517 cases have been treated in the intern department of this institution.

At the festival dinner of the East London Hospital for Children, it was stated that the institution was earnestly in want of money, nearly £1,500 being required to clear off the debt incurred in connection with the out-patient department. As a result of the appeal, subscriptions and donations close upon £1,500 were now announced.

A committee of gentlemen has for some time been trying to establish a convalescent home for the fever patients of both the borough and workhouse hospitals at Sunderland. They have now secured, at the rent of £19 a year, a cottage, which they have taken for twelve months as an experiment, relying upon the support of the public.

A bazaar in aid of the Manchester Southern Hospital for Women and Children was held last week in the new buildings of the Medical School of Owens College. A scheme has been arranged for the amalgamation of St. Mary's Hospital with the Southern, and under certain conditions as to this amalgamation the offer of £70,000 has been made by the trustees of the Davis Lewis Trust.

Their Royal Highnesses Princess Christian and Princess Mary Duchess of Teck, have graciously consented to be patronesses of the entertainment to be given in the Chelsea Town Hall on the 21st inst. by Mr. Cyril Maude, Miss Winifred Emery, and other artistes in aid of the funds of the Grosvenor Hospital for Women and Children, Vincent Square, Westminster.

### Medical Vacancies and Appointments.

### VACANCIES.

**Resident Medical Officers.**

Flintshire Dispensary.—Resident House Surgeon. Salary £120 per annum, with furnished house (rent and taxes free), and coal, light, water, and cleaning, or in lieu thereof £20 per annum ; knowledge of Welsh desirable. Applications to W. T. Cole, Secretary, Board Room, Bangillt Street, Holywell, by June 5th.

Glamorganshire and Monmouthshire Infirmary, Cardiff.—Assistant Resident Medical Officer. Appointment for six months. No salary, but board, washing, and apartments. Applications and testimonials to G. T. Colman, Secretary, by June 9th.

North Staffordshire Infirmary and Eye Hospital, Hartshill, Stoke-upon-Trent.—Honorary Assistant House Surgeon. Applications to the Secretary by June 5th.

Public Dispensary, 59, Stanhope Street, Clare Market.—Resident Medical Officer. Salary £105 per annum, with furnished apartments, coals, and gas. Applications to the Secretary by June 8th.

Royal Free Hosptial, Gray's Inn Road, W.C.—Two Junior Resident Medical Officers; doubly qualified. No salary; board, lodging, and washing provided. Applications to the Secretary by June 2nd.

**Non-Resident Medical Officers.**

Glasgow Maternity Hospital, 146, Buchanan Street, Glasgow.—Indoor and Outdoor Surgeons. Applications to Arthur Forbes, Secretary, by June 9th.

Hospital for Epilepsy and Paralysis and other Diseases of the Nervous System, 32, Portland Terrace, Regent's Park, N.W.—Physician to Out-patients. Applications to the Secretary by June 8th.

Westminster Hospital, Broad Sanctuary, S.W.—Dental Surgeon. Duly qualified and registered. Must attend the House Committee on Tuesday, June 5th.

The Yorkshire College, Leeds, Department of Medicine.—Honorary Demonstrator of Surgical Pathology. Applications to the Secretary by May 31st.

